# A New Determinant of *Candida glabrata* Virulence: The Acetate Exporter CgDtr1

**DOI:** 10.3389/fcimb.2017.00473

**Published:** 2017-11-14

**Authors:** Daniela Romão, Mafalda Cavalheiro, Dalila Mil-Homens, Rui Santos, Pedro Pais, Catarina Costa, Azusa Takahashi-Nakaguchi, Arsénio M. Fialho, Hiroji Chibana, Miguel C. Teixeira

**Affiliations:** ^1^Biological Sciences Research Group, Department of Bioengineering, Instituto Superior Técnico, Universidade de Lisboa, Lisbon, Portugal; ^2^Institute for Bioengineering and Biosciences, Biological Sciences Research Group, Instituto Superior Técnico, Lisbon, Portugal; ^3^Medical Mycology Research Center, Chiba University, Chiba, Japan

**Keywords:** *Candida glabrata*, CgDtr1, virulence, *Galleria mellonella*, acetic acid resistance

## Abstract

Persistence and virulence of *Candida glabrata* infections are multifactorial phenomena, whose understanding is crucial to design more suitable therapeutic strategies. In this study, the putative multidrug transporter CgDtr1, encoded by ORF *CAGL0M06281g*, is identified as a determinant of *C. glabrata* virulence in the infection model *Galleria mellonella*. *CgDTR1* deletion is shown to decrease the ability to kill *G. mellonella* larvae by decreasing *C. glabrata* ability to proliferate in *G. mellonella* hemolymph, and to tolerate the action of hemocytes. The possible role of CgDtr1 in the resistance to several stress factors that underlie death induced by phagocytosis was assessed. *CgDTR1* was found to confer resistance to oxidative and acetic acid stress. Consistently, CgDtr1 was found to be a plasma membrane acetic acid exporter, relieving the stress induced upon *C. glabrata* cells within hemocytes, and thus enabling increased proliferation and virulence against *G. mellonella* larvae.

## Introduction

Infections caused by *Candida* species are recognized as the 4th most common cause of nosocomial infections (Wisplinghoff et al., 2004). Candidiasis is considered to be responsible for more than 400,000 life-threatening infections worldwide every year (Denning and Bromley, 2015). *Candida glabrata* is the second most common cause of invasive candidiasis, with an estimated death rate of 40–50% (Tscherner et al., 2011). Infections caused by this pathogenic yeast have increased worldwide (Rodrigues et al., 2014; Yapar, 2014), which is likely due to the relatively high prevalence of antifungal drug resistance among *C. glabrata* clinical isolates (Tscherner et al., 2011; Pfaller et al., 2014). It may also be the result of its ability to survive under stress conditions, such as those imposed by the host immune system (Pfaller et al., 2014). Additionally, *C. glabrata* is able to survive on inanimate surfaces for more than 5 months, while *C. albicans* can only sustain about 4 months and *C. parapsilosis* only 2 weeks (Kramer et al., 2006). It is, thus, crucial to fully understand these pathogenesis-related phenomena in order to design better ways to prevent and control superficial and invasive candidiasis caused by *C. glabrata*.

In this work, the predicted *C. glabrata* Drug:H^+^ antiporter (DHA) CgDtr1, encoded by the ORF *CAGL0M06281g*, is shown to play a role in virulence and biofilm formation. ORF *CAGL0M06281g* is predicted to encode an ortholog of the *S. cerevisiae* Dtr1 protein, which was characterized as a dityrosine transporter required for spore wall synthesis (Felder et al., 2002). Dtr1 was further shown to confer resistance to the antimalarial drug quinine, the antiarrhythmic drug quinidine and weak acids (Felder et al., 2002). Within the DHA family in *C. glabrata*, seven other homologous transporters have been characterized so far (Costa et al., 2014a), and found to confer resistance to drugs and other stress factors, such as azoles, flucytosine, benomyl, acetic acid, and polyamines, mostly by contributing to decrease the intracellular accumulation of those molecules (Chen et al., 2007; Costa et al., 2013a,b, 2014b; Pais et al., 2016a,b). Interestingly, the *C. albicans* DHA transporters CaMdr1, CaNag3, CaNag4 and, more recently, CaQdr1, CaQdr2, and CaQdr3 were found to further play a role in its virulence, although the molecular mechanisms behind this observation are still to be fully elucidated (Becker et al., 1995; Yamada-Okabe and Yamada-Okabe, 2002; Shah et al., 2014). More recently, the DHA transporters CgTpo1_1 and CgTpo1_2 were also found to contribute to increase *C. glabrata* virulence, through increasing antimicrobial peptide resistance and tolerance against phagocytosis, respectively (Santos et al., 2017).

In this study, the impact of CgDtr1 expression in the context of virulence was studied, using *Galleria mellonella* as an infection model. The influence of CgDtr1 in *C. glabrata* proliferation in this host and in the presence of *G. mellonella* hemocytes was further evaluated. Additionally, the activity of CgDtr1 against phagocytosis-related stresses, including oxidative and acidic stress, was inspected.

## Methods

### Strains, plasmids, and growth medium

*Candida glabrata* CBS138 (prototrophic), KUE100 (prototrophic) (Ueno et al., 2007) and L5U1 (cgura3Δ0; cgleu2Δ0) (Chen et al., 2007) strains were used in this study. *C. glabrata* cells were cultivated in rich YEPD medium, RPMI 1640 medium or BM minimal medium. YEPD medium contained per liter: 20 g D-(+)- glucose (Merk), 20 g bacterial-peptone (LioChem) and 10 g of yeast-extract (Difco); whereas RPMI 1640 medium (pH 4) contained 10.4 g RPMI 1640 (Sigma), 34.5 g MOPS (Sigma) and 18 g glucose (Merck) per liter. BM media contained 20 g/L of D-(+)-glucose (Merck), 1.7 g/L of Yeast-Nitrogen-Base, without amino acids or ammonium sulfate (Difco) and 2.65 g/L ammonium sulfate (Merck). L5U1 strain was grown in BM medium supplemented with 60 mg/L leucine (Sigma). *Saccharomyces cerevisiae* strain BY4741 (*MAT*a, ura3Δ0, leu2Δ0, his3Δ1, met15Δ0) and the derived single deletion mutant BY4741_Δ*dtr1* were obtained from the Euroscarf collection and grown in BM medium supplemented with 20 mg/L methionine, 20 mg/L histidine, 60 mg/L leucine, and 20 mg/L uracil (all from Sigma). Solid media contained additionally 20 g/L of Bactoagar (Difco).

### Cloning of the *C. glabrata CgDTR1* gene (ORF CAGL0M06281g)

The pGREG576 plasmid from the Drag & Drop collection (Jansen et al., 2005) was used to clone and express the *C. glabrata* ORF *CAGL0M06281g* in *S. cerevisiae*, as described before (Costa et al., 2013b). *CAGL0M06281g* DNA was generated by PCR, using genomic DNA extracted from the sequenced CBS138 *C. glabrata* strain, and the following specific primers: 5′-GAATTCGATATCAAGCTTATCGATACCGTCGACA*ATGAGCACCTCCAGCAACAC*-3′ and 5′-GCGTGACATAACTAATTACATGACTCGAGGTCGAC*TCAGAAACTGTCTTTAACCC*-3′. The designed primers contain, besides a region with homology to the first and last 20 nucleotides of the *CAGL0M06281g* coding region (italic), nucleotide sequences with homology to the cloning site flanking regions of the pGREG576 vector (underlined). The amplified fragment was co-transformed into the parental *S. cerevisiae* strain BY4741 with the pGREG576 vector, previously cut with the restriction enzyme SalI, to obtain the pGREG576_*CgDTR1* plasmid. To enable gene expression in *C. glabrata*, the *GAL1* promoter present in the pGREG576_*CgDTR1* plasmid was replaced by the copper-inducible *C. glabrata MTI* promoter, giving rise to the pGREG576_MTI_*CgDTR1* plasmid. The *MTI* promoter DNA was generated by PCR with the specific primers: 5′-TTAACCCTCACTAAAGGGAACAAAAGCTGGAGCTC*TGTACGACACGCATCATGTGGCAATC*-3′ and 5′-GAAAAGTTCTTCTCCTTTACTCATACTAGTGCGGC*TGTGTTTGTTTTTGTATGTGTTTGTTG*-3′. The designed primers contain, besides a region with homology to the first and last 27 nucleotides of the first 1,000 bp of the *MTI* promoter region (italic), nucleotide sequences with homology to the cloning site flanking regions of the pGREG576 vector (underlined). The amplified fragment was co-transformed into the parental strain BY4741 with the pGREG576_*CgDTR1* plasmid, previously cut with SacI and NotI restriction enzymes to remove the *GAL1* promoter, to generate the pGREG576_*MTI*_*CgDTR1* plasmid. The recombinant plasmids pGREG576_*CgDTR1* and pGREG576_*MTI*_*CgDTR1* were verified by DNA sequencing.

### Disruption of the *C. glabrata CgDTR1* gene (ORF *CAGL0M06281g*)

The deletion of the *CgDTR1* gene was carried out in the parental strain KUE100, as described before (Ueno et al., 2007). The target genes were replaced by a DNA cassette including the CgHIS3 gene, through homologous recombination. The replacement cassette was prepared by PCR using the following primers: 5′-TTTTGATTTTTTTACCATAATTTGTTTAAGATTATTTACCATAATAGCAGTAACGG*GGCCGCTGATCACG*-3′ and 5′-ACACTAAATTTTAAACTTAGAATTCATTGAAGGCCCCTTAGAAATTATAAGTTTCA*CATCGTGAGGCTGG*-3′. The pHIS906 plasmid including *CgHIS3* was used as a template and transformation was performed as described previously. The 3′ sequences of the primers, GGCCGCTGATCACG and CATCGTGAGGCTGG were attached to flanking region of *CgHIS3*. The underlined 56 bp sequences were flanking sequence of the each gene. The replacement of *CgDTR1* by the *CgHIS3* cassette was verified by PCR, using as primers: 5′-CAGCTTTATCTCAGAAAACCAG-3′, which is complementary to a region in the cassette; and 5′-GCTAAGAGATTGGCTGAGAG-3′, which is complementary to a region downstream of the *CgDTR1* 3′ end. Additionally, gene deletion was further verified by PCR using the following pairs of primers: 5′-*CAACACTAGCGGTGAGTG*-3′ and 5′-*CTGCCTCCTTTATCTGCGA*-3′. No PCR product was identified from template DNA of the mutant, while clear PCR products were identified from template DNA of parental strain KUE100.

### *Galleria mellonella* survival and proliferation assays

*Galleria mellonella* larvae were reared in our lab insectariums, on a pollen grain and bee wax diet at 25°C in the darkness. Last instance larvae weighting 250 ± 25 mg were used in the killing assays and the larvae infection was based on the protocol previously described (Cotter et al., 2000; Mil-Homens and Fialho, 2012; Santos et al., 2017). *C. glabrata* strains were cultivated in YEPD until stationary phase, harvested by centrifugation and resuspended in PBS (pH 7.4). 3.5 μL of yeast cell suspension, containing ~5 × 10^7^ cells, were injected into each caterpillar via the last left proleg. For each condition, 10 larvae were used to follow the larval survival over a period of 72 h. Control larvae were injected with PBS (pH 7.4). For proliferation assays, three living larvae at 1, 24, and 48 h post-injection were punctured in the abdomen with a sterile needle and hemolymph was collected.

### *C. glabrata* phagocytosis assays in *G. mellonella* hemocytes

*G. mellonella* hemocytes were isolated as described before (Brivio et al., 2010). Hemocytes suspended in Grace insect medium (GIM) (Sigma) supplemented with 10% fetal bovine serum, 1% glutamine, and 1% antibiotic (10,000 U penicillin G, 10 mg streptomycin).

Cultures of *C. glabrata* cells were grown until mid-exponential phase (OD600 nm = 0.4–0.6) and the appropriate volume was collected to have 7 × 10^2^ cells/ml in PBS. *Galleria* hemocyte monolayer medium was replaced with GIM without antimycotics, and then cells were infected with the yeast suspensions with a multiplicity of infection (MOI) of 1:5. After 1 h of infection at 37°C, the hemocytes were carefully washed twice with cell culture medium. Viable intracellular yeast cells were measured after 1, 4, 24, and 48 h of infection, upon hemocyte lysis with 0.5% Triton X-100.

### Susceptibility assays in *C. glabrata* and *S. cerevisiae*

The susceptibility of the *C. glabrata* or *S. cerevisiae* cells toward toxic concentrations of the selected drugs and acetic acid was evaluated as before, through spot assays or cultivation in liquid growth medium (Costa et al., 2013a,b). The tested drugs included the following compounds, used in the specified concentration ranges: the azole antifungal drugs fluconazole (10–200 mg/l), ketoconazole (10–50 mg/l), clotrimazole (1–20 mg/l), thioconazole (0.2–1 mg/l) and miconazole (0.2–1 mg/l), the polyene antifungal drug amphotericin B (0.1–0.5 mg/l), and the fluoropyrimidine 5-flucytosine (0.02–5 mg/l).

### CgDTR1 sub-cellular localization assessment

The sub-cellular localization of the CgDtr1 protein was determined based on the observation of BY4741 *S. cerevisiae* or L5U1 *C. glabrata* cells transformed with the pGREG576-*CgDTR1* or pGREG576-MTI-*CgDTR1* plasmids, respectively. *S. cerevisiae* cell suspensions were prepared by cultivation in BM-U medium, containing 0.5% glucose and 0.1% galactose, at 30°C, with orbital shaking (250 rev/min), until a standard culture OD600 nm (Optical Density at 600 nm) = 0.4 ± 0.04 was reached. At this point cells were transferred to the same medium containing 0.1% glucose and 1% galactose, to induce protein expression. *C. glabrata* cell suspensions were prepared in BM-U medium, until a standard culture OD600 nm = 0.5 ± 0.05 was reached, and transferred to the same medium supplemented with 50 μM CuSO4 (Sigma), to induce protein over-expression. After 5 h of incubation, the distribution of CgDtr1_GFP fusion protein in *S. cerevisiae* or in *C. glabrata* living cells were detected by fluorescence microscopy in a Zeiss Axioplan microscope (Carl Zeiss MicroImaging), using excitation and emission wavelength of 395 and 509 nm, respectively, and images captured using a cooled CCD camera (Cool SNAPFX, Roper Scientific Photometrics).

### ^14^C-acetate accumulation assay

Accumulation assays using radiolabelled, [^14^C]-acetate were carried out as described before (Costa et al., 2013a). To estimate the accumulation of radiolabelled compound (Intracellular/Extracellular) in yeast cells, cells were grown in BM medium, resuspended in BM medium, to obtain dense cell suspensions (OD600 nm = 5.0 ± 0.1, equivalent to ~2.2 mg/(dry weight) ml). The radiolabeled compound was added to the cell suspensions (3.5 μM of [^14^C]-acetic acid (American Radiolabeled Chemicals; 0.1 mCi/ml) and 65 mM of unlabeled acetic acid) and accumulation of radiolabeled compound was followed for 30 min until equilibrium was reached. Radioactivity was measured in a Beckman LS 5000TD scintillation counter.

### Gene expression measurement

The transcript levels of *CgDTR1* were determined by quantitative real-time PCR (RT-PCR). Total RNA was extracted from cells grown in the presence of acetic acid or hydrogen peroxide, and also when internalized within larvae hemocytes. At the end of each incubation period, total RNA obtained from the *C. glabrata* cell population was extracted using the hot phenol method (Köhrer and Domdey, 1991). Total RNA was converted to cDNA for the real-time Reverse-Transcription PCR (RT-PCR) using the MultiScribe Reverse Transcriptase kit (Applied Biosystems) and the 7500 RT-PCR thermal cycler block (Applied Biosystems). The real time PCR step was carried out using adequate primers (*CgDTR1* Forward: 5′-GGAGCCAAAATGAGAATGATATGTC−3′; *CgDTR1* Reverse: 5′-ACCACCTTGAAATCGGTGATG−3′; *CgACT1* Forward: 5′-AGAGCCGTCTTCCCTTCCAT-3′; *CgACT1* Reverse: 5′-TTGACCCATACCGACCATGA-3′), SYBR Green® reagents (Applied Biosystems) and the 7500 RT-PCR thermocycler block (Applied Biosystems). Default parameters set by the manufacturer were followed, and fluorescence was detected by the instrument and plotted in an amplification graph (7500 Systems SDS Software, Applied Biosystems). *CgACT1* gene transcript level was used as an internal reference. To avoid false positive signals, the absence of non-specific amplification with the chosen primers was confirmed by the generation of a dissociation curve for each pair of primers.

## Results

### CgDtr1 is a determinant of *C. glabrata* virulence against the *G. mellonella* infection model

**Figure 1:**
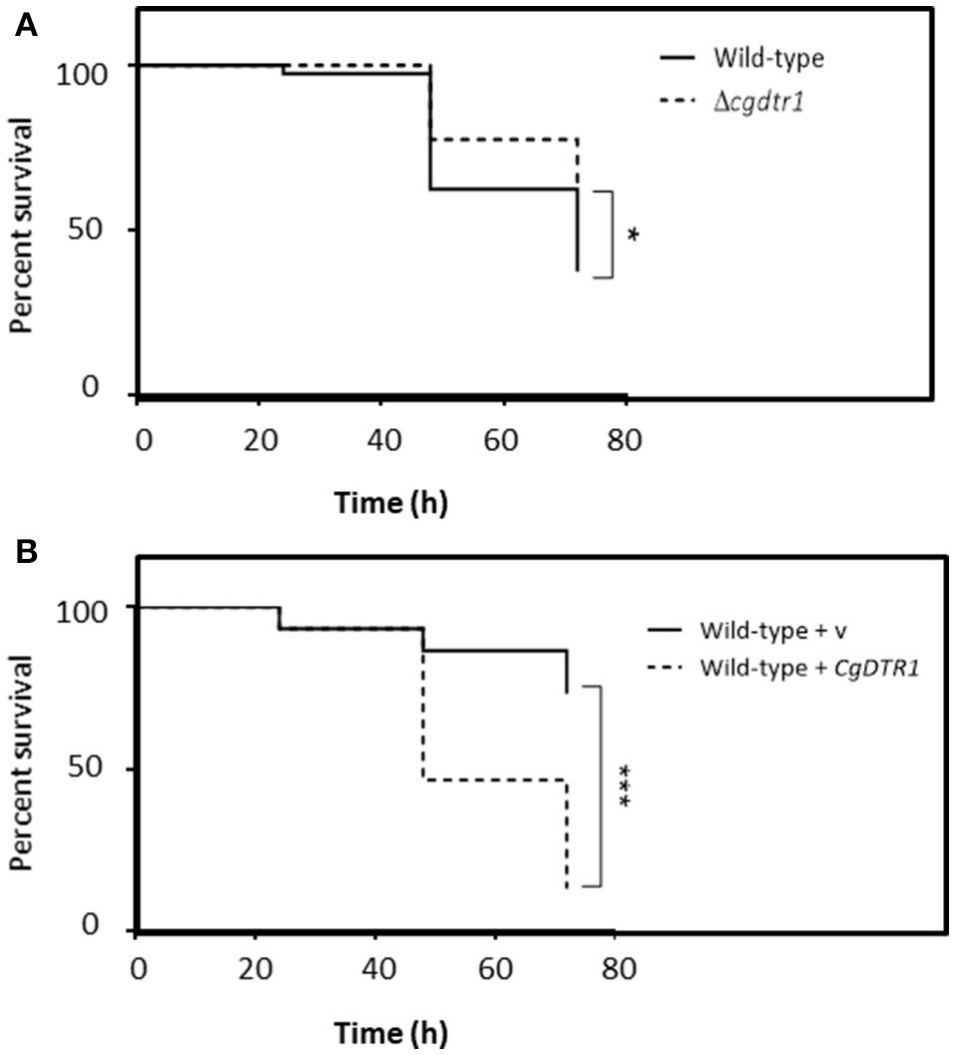
*CgDTR1* expression increases *C. glabrata* virulence against the *G. mellonella* infection model. **(A)** The survival of larvae injected with ~5 × 10^7^ CFU/larvae of KUE100 *C. glabrata* wild-type (full line), or derived Δ*cgdtr1* deletion mutant (dashed line), is displayed as Kaplan-Meier survival curves. **(B)** The survival of larvae injected with ~5 × 10^7^ CFU/larvae of L5U1 *C. glabrata* wild-type strain, harboring the pGREG576 cloning vector (full line) or the pGREG576_MTI_*CgDTR1* expression plasmid (dashed line), is displayed as Kaplan-Meier survival curves. The displayed results are the average of at least three independent experiments. ^*^P < 0.05; ^***^P < 0.001.

Using *G. mellonella* larvae as an infection model, the possible effect of *CgDTR1* deletion in *C. glabrata* ability to exert its virulence was assessed. The survival of the larvae was followed for 72 h upon injection of 5 × 10^7^ cells. The wild-type KUE100 *C. glabrata* strain was found to be able to kill 30% more larvae than the derived Δ*cgdtr1* deletion mutant (Figure 1A). Additionally, the over-expression of *CgDTR1* in the L5U1 *C. glabrata* wild-type was found to lead to a 50% decrease in *G. mellonella* survival rate, when compared with L5U1 cells harboring the cloning vector pGREG576 (Figure 1B). Interestingly, it was also possible to observe that the L5U1 wild-type strain appears to be much less virulent than the KUE100 wild-type strain. This data strongly suggest that the CgDtr1 transporter is involved in the pathogenesis of *C. glabrata*.

### *CgDTR1* expression increases *C. glabrata* cell proliferation in *G. mellonella* hemolymph

**Figure 2:**
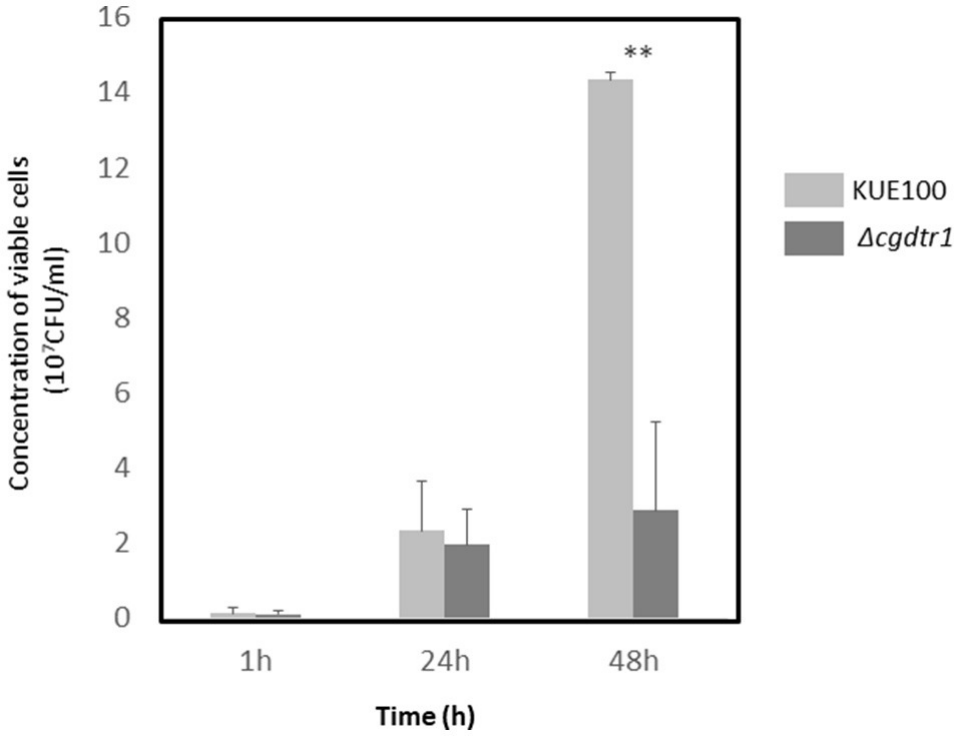
*CgDTR1* deletion decreases *C. glabrata* proliferation in *G. mellonella* hemolymph. The concentration of viable *C. glabrata* KUE100 wild-type (light gray) and Δ*cgdtr1* (dark gray) cells assessed within total hemolymph recovered from *G. mellonella* larvae upon 1, 24, and 48 h of injection with ~5 × 10^7^ CFU/larvae is shown. The displayed results are the average of at least three independent experiments, standard deviation being represented by the error bars. ^**^P < 0.01.

Given the observation that CgDtr1 expression increases the ability of *C. glabrata* cells to kill *G. mellonella* larvae, a deeper analysis of the underlying mechanisms was undertaken. The proliferation of *C. glabrata* cells within this infection model was then assessed. The hemolymph of injected larvae was recovered 1, 24, and 48 h after injection and the number of viable *C. glabrata* cells evaluated. After 1 and 24 h of injection the number of wild-type and Δ*cgdtr1* cells was found to be undistinguishable (Figure 2). However, upon 48 h of injection, a time in which a greater difference was detected in terms of larvae survival rate, the concentration of wild-type cells was found to be 4.5-fold higher than that of the mutant population (Figure 2). This difference in terms of cell proliferation may very well explain the different ability to kill the host, especially considering that the mutant displays no growth defect *in vitro*.

**Figure 3:**
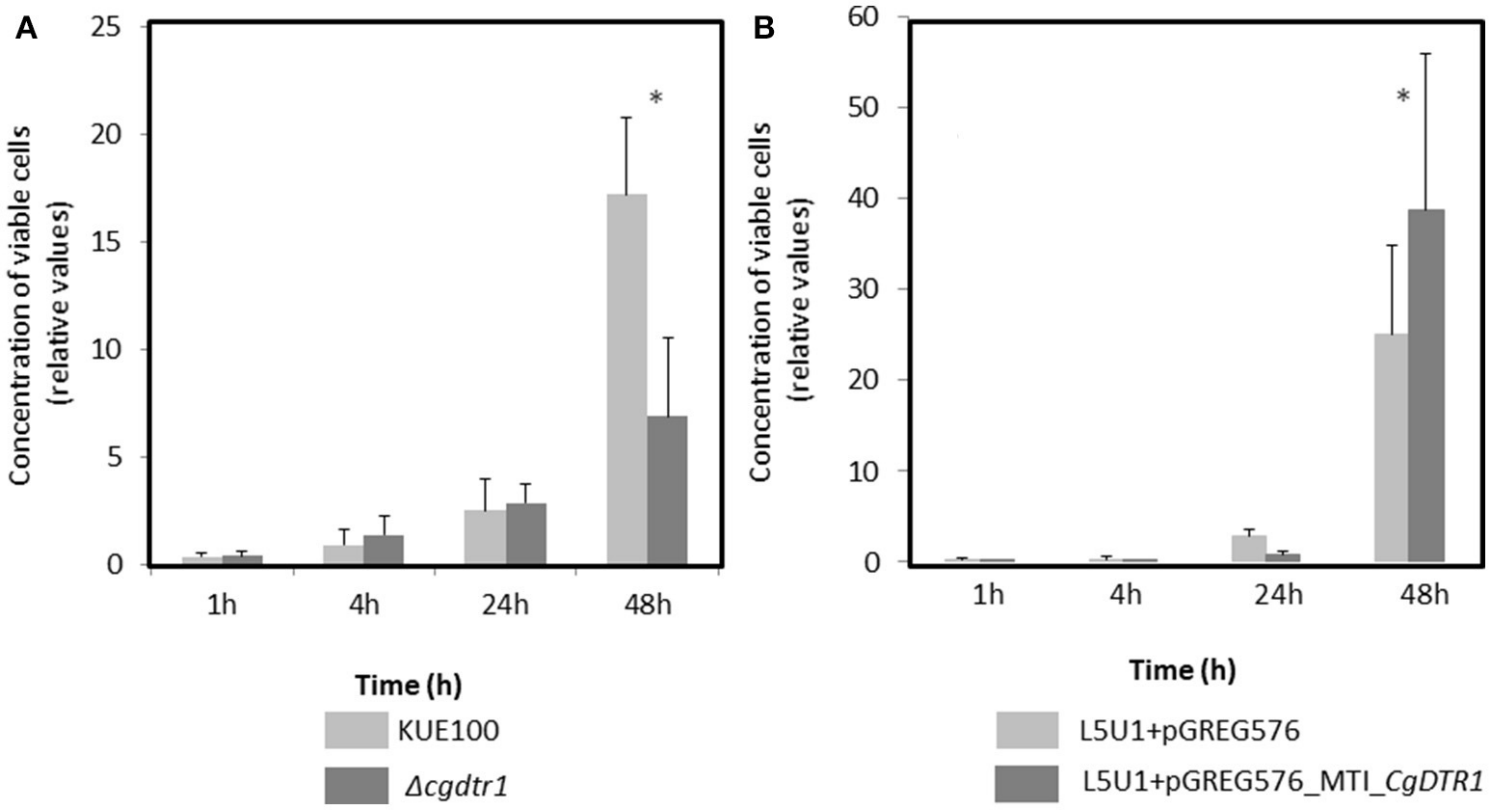
*CgDTR1* expression increases *C. glabrata* proliferation in *G. mellonella* hemocytes. The concentration of viable KUE100 *C. glabrata* wild-type (light gray) and Δ*cgdtr1* (dark gray) cells **(A)**, or of viable L5U1 *C. glabrata* wild-type harboring the pGREG576 cloning vector (light gray) or the pGREG576_MTI_*CgDTR1* expression plasmid (dark gray) cells **(B)** assessed upon 1, 4, 24, and 48 h of co-culture with *G. mellonella* hemocytes, using a MOI of 1:5. The displayed results are relative to the concentration of viable cells inoculated at time zero and are the average of at least three independent experiments, standard deviation being represented by the error bars. ^*^P < 0.05.

The exact reason why the Δ*cgdtr1* mutant is unable to proliferate as much as the wild-type within the *G. mellonella* hemolymph was then pursued. First, the hypothesis that the two strain exhibit differences at the level of antimicrobial peptide (AMP) resistance was explored. The resistance of both strains to the AMP histatine-5 was evaluated, however, no differences were observed (results not shown). Second, the resistance of each strain to phagocytosis by hemocytes, the insect equivalent to mammalian macrophages, was inspected. *C. glabrata* cells were exposed to a cell culture of *G. mellonella* hemocytes. The concentration of viable *C. glabrata* cells found within the hemocytes was measured after 1, 4, 24, and 48 h after co-culture. After 1, 4, and 24 h of phagocytosis the number of viable wild-type cells internalized within hemocytes was found to be undistinguishable of the number of Δ*cgdtr1* cells internalized within hemocytes (Figure 3A). After 48 h of injection, the population of wild-type cells found within hemocytic cells was found to be 3-fold higher than Δ*cgdtr1* mutant cells (Figure 3A). A similar experiment was carried out using the L5U1 *C. glabrata* wild-type cells harboring the pGREG576_MTI_*CgDTR1*, leading to *CgDTR1* over-expression upon CuSO_4_ induction, or the cloning vector pGREG576. Consistent with the previous results, the over-expression of *CgDTR1* was found to lead to a 25% increase in cell proliferation within hemocytes (Figure 3B).

### CgDtr1 confers resistance to weak acid and oxidative stress, but not to antifungal drugs

**Figure 4:**
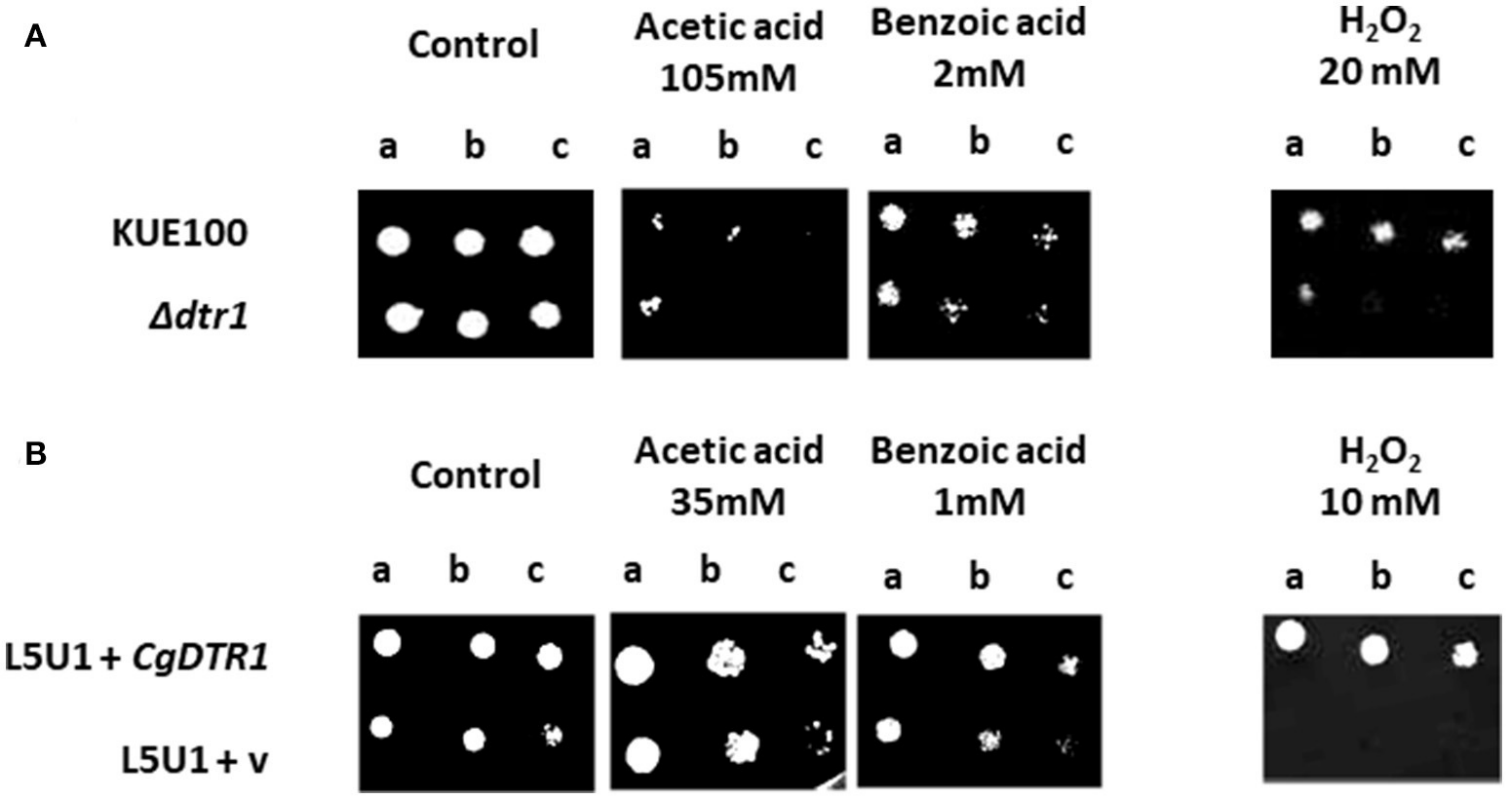
*CgDTR1* confers resistance to weak acids and hydrogen peroxide in *C. glabrata*. **(A)** Comparison of the susceptibility to acetic acid, benzoic acid or to H_2_O_2_, at the indicated concentrations, of the KUE100 *C. glabrata* (wild-type—wt) and Δ*cgdtr1* strains, in BM agar plates, pH 4.5, by spot assays. **(B)** Comparison of the susceptibility to acetic acid, benzoic acid or to H_2_O_2_, at the indicated concentrations, of the L5U1 *C. glabrata* strain (wild-type—wt), harboring the pGREG576 cloning vector (v) or the pGREG576_MTI_*CgDTR1* in BM agar plates, pH 4.5, without uracil, by spot assays. The inocula were prepared as described in the materials and methods section. Cell suspensions used to prepare the spots were 1:5 (b) and 1:25 (c) dilutions of the cell suspension used in (a). The displayed images are representative of at least three independent experiments.

**Figure 5:**
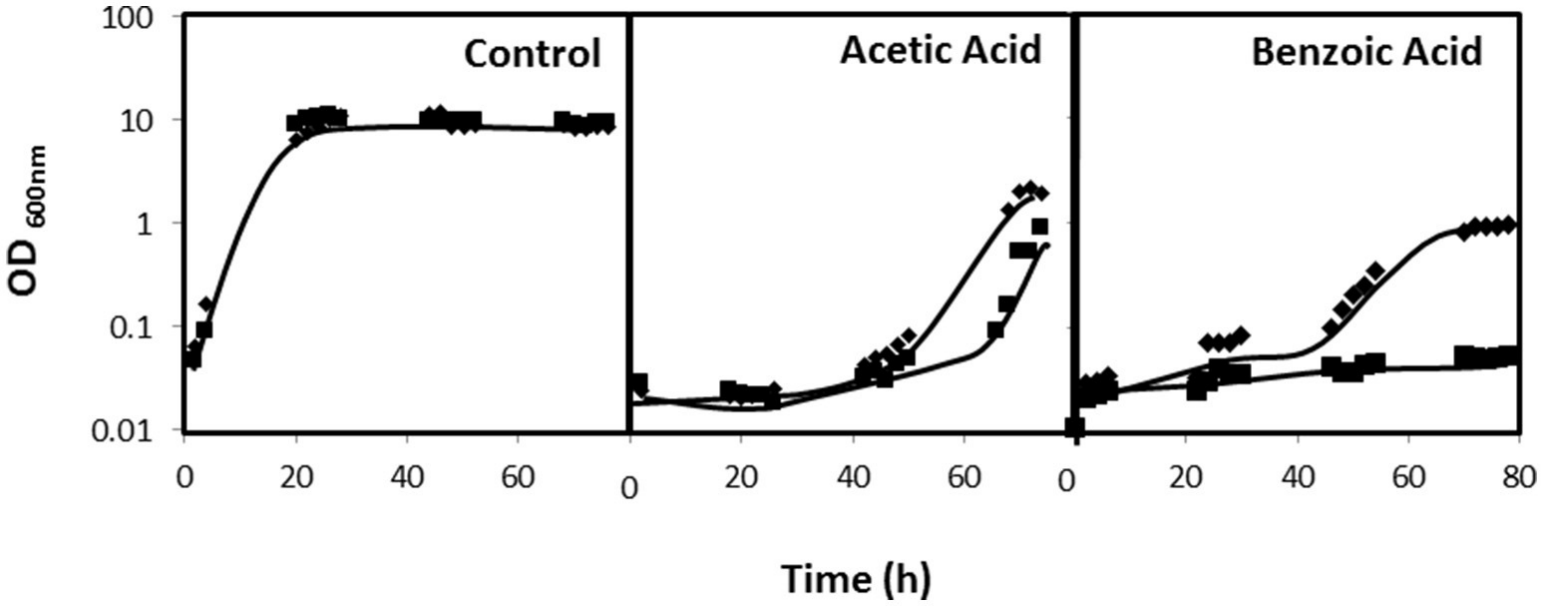
*CgDTR1* confers resistance to weak acids in *C. glabrata*. Comparison of the susceptibility of KUE100 *C. glabrata* wild-type (♦) and Δ*cgdtr1* (■) deletion mutant strain to cultivation in the presence of 100 mM acetic acid or of 2 mM benzoic acid in BM liquid medium, pH 4.5, in comparison to control conditions, measured in terms of variation of Optical Density at 600nm (OD600nm). The inocula were prepared as described in the materials and methods section. Growth curves are representative of at least three independent experiments.

Given its predicted function as a multidrug resistance transporter, together with the results described above, it appeared reasonable to hypothesize that the role of CgDtr1 in *C. glabrata* proliferation within *G. mellonella* hemocytes is related to defending the yeast cell against the stress factors encountered within hemocytic cells. Considering the identification of the *S. cerevisiae DTR1* gene as a determinant of weak acid resistance, the eventual role of *CgDTR1* in this context was evaluated, especially considering that the phagolysosomes of mammalian macrophages are highly acidic and rich in acetic acid (Peleg et al., 2010). Based on spot assays, the deletion of *CgDTR1* was found to moderately decrease acetic and benzoic acid tolerance in the KUE100 *C. glabrata* strain (Figure 4A). Given the importance of Reactive Oxygen Species (ROS) in the killing of phagocytosed yeast cells, the effect of the expression of *CgDTR1* in the resistance to hydrogen peroxide was further assessed. Significantly, the deletion of *CgDTR1* was also found to severely impair growth in the presence of 20 mM H_2_O_2_ (Figure 4A). Based on the analysis of the growth curves of the same populations in liquid medium, it is possible to see that the deletion of the *C. glabrata CgDTR1* gene dramatically increases yeast susceptibility to acetic acid, increasing in about 20 h the period of lag-phase induced upon stress exposure (Figure 5). Upon exposure to 2 mM benzoic acid, wild-type cells were found to undergo a period of 40 h of lag-phase, before exponential growth could be resumed, but in these conditions the Δ*cgdtr1* deletion mutant population was found to be unable to resume growth even after 80 h of lag-phase (Figure 5). Consistent with the previous results, the over-expression of the *CgDTR1* gene in the L5U1 *C. glabrata* wild-type strain was found to increase its resistance to the acetic and benzoic acid stress, as well as to H_2_O_2_ (Figure 4B). Interestingly, it was also possible to observe that the L5U1 wild-type strain appears to be much more susceptible to weak acid and oxidative stress than the KUE100 wild-type strain. Furthermore, the heterologous expression of the *CgDTR1* gene was found to slightly increase the resistance of the *S. cerevisiae* wild-type and Δ*dtr1*deletion mutant strains against both weak acids and H_2_O_2_ (results not shown).

Given the predicted role of CgDtr1, as a member of the drug:H^+^ antiporter family, in drug/chemical stress resistance, the susceptibility to antifungal drugs was additionally assessed. The tested drugs include the azoles miconazole, thioconazole, clotrimazole, ketoconazole, itraconazole, and fluconazole, the polyene amphotericin B, and the fluoropyrimidine 5-flucytosine. However, no difference in terms of drug susceptibility was observed when comparing the proliferation of wild-type and Δ*cgdtr1* deletion mutant strains (results not shown).

### *CgDTR1* is a plasma membrane acetate exporter

**Figure 6:**
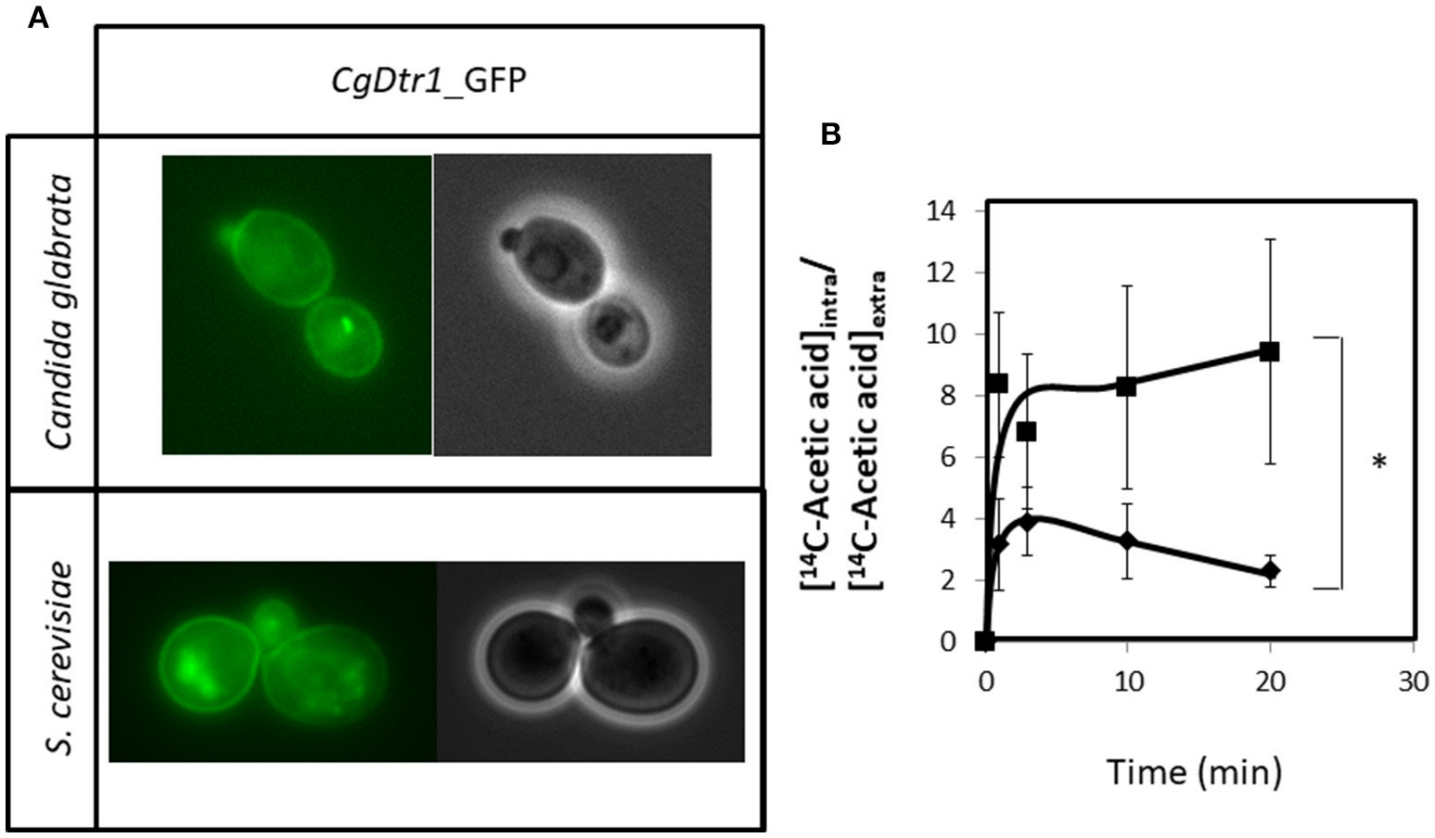
CgDtr1 is a plasma membrane acetate exporter in *C. glabrata* cells. **(A)** Fluorescence of exponential-phase L5U1 *C. glabrata* cells or BY4741 *S. cerevisiae* cells, harboring the pGREG576_MTI_*CgDTR1* or pGREG576_*CgDTR1* plasmids, after 5 h of copper- or galactose-induced recombinant protein production, respectively. Results indicate that the CgDtr1-GFP fusion protein localizes to the plasma membrane of both *S. cerevisiae* and *C. glabrata* cells. **(B)** Time course accumulation ratio of [^14^C]-Acetic acid in non-adapted cells of KUE100 *C. glabrata* wild-type (♦) or Δ*cgdtr1* (■) strains, during cultivation in BM liquid medium in the presence of 65 mM unlabeled acetic acid. The accumulation ratio values are averages of at least three independent experiments. Error bars represent the corresponding standard deviations. ^*^p < 0.05.

*C. glabrata* cells harboring the pGREG576_MTI_*CgDTR1* plasmid were grown to mid-exponential phase in minimal medium, and then transferred to the same medium containing 50 μM CuSO_4_, to induce the expression of the fusion protein. At a standard OD_600nm_ of 0.5 ± 0.05, obtained after around 5 h of incubation, cells were inspected through fluorescence microscopy. In *C. glabrata* cells, the CgDtr1_GFP fusion protein was found to be predominantly localized to the cell periphery (Figure 6A). *S. cerevisiae* cells harboring the pGREG576_*CgDTR1* plasmid were also tested for the subcellular localization of CgDtr1, to verify that in these cells, the *C. glabrata* transporter was localized similarly. At a standard OD_600nm_ of 0.5 ± 0.05, obtained after around 5 h of incubation with 1% galactose to induce protein expression, fluorescence was found mostly in the cell periphery (Figure 6A). Intracellular Dtr1-GFP staining is also observed, possibly corresponding to mislocalized protein aggregates resulting from exaggerated over-expression. Altogether, these results strongly suggest plasma membrane localization, differently from what was observed for its *S. cerevisiae* homolog, Dtr1, shown to be localized only at the prospore membrane (12).

Given the registered plasma membrane localization, the possible role of CgDtr1 as an acetate exporter was analyzed. The accumulation of [^14^C]-labeled acetic acid in non-adapted *C. glabrata* cells suddenly exposed to the presence of 65 mM cold acetic acid, which induces a mild growth inhibition in both the parental strain and Δ*cgdtr1* cells, was thus tested. The intracellular accumulation of radiolabelled acetate was found to be 2-fold higher in cells devoid of *CgDTR1* than in parental KUE100 cells (Figure 6B). Altogether, the presented evidences point out to CgDtr1 as a plasma membrane acetate exporter.

### *CgDTR1* transcript levels are up-regulated during internalization in hemocytes and in the presence of hydrogen peroxide stress

**Figure 7:**
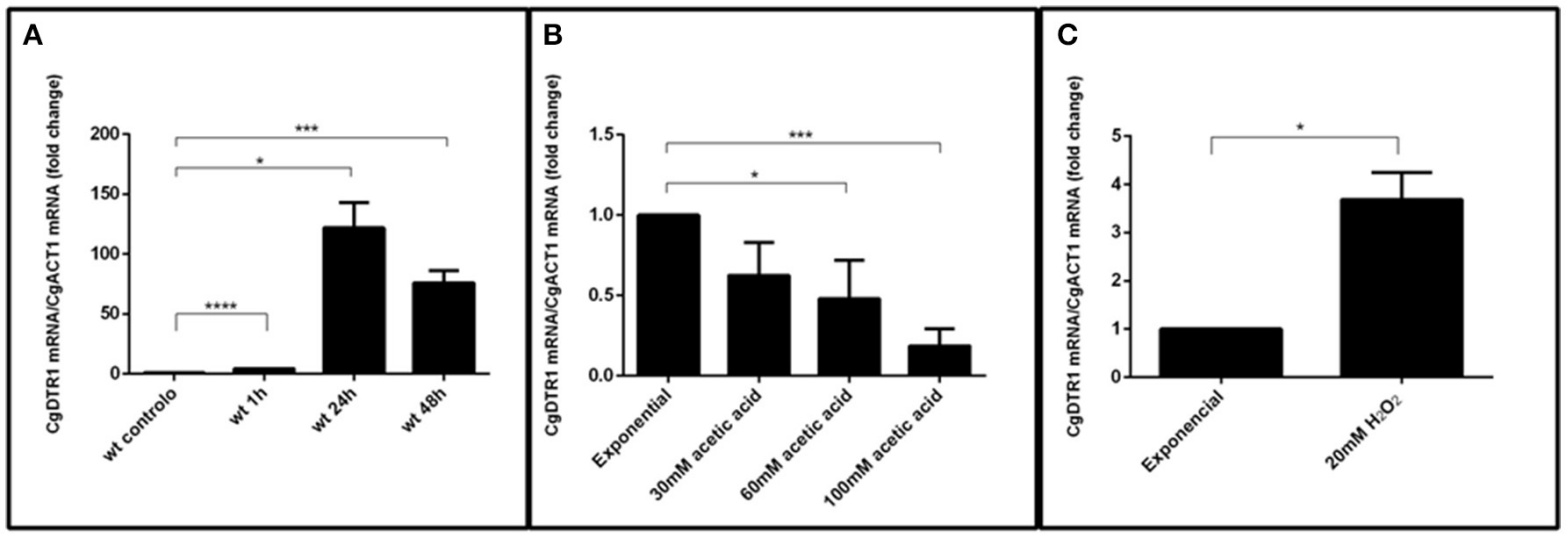
The *CgDTR1* gene transcript level is up-regulated during internalization in hemocytes and upon exposure to oxidative stress. Comparison of the variation of the *CgDTR1* transcript levels in KUE100 *C. glabrata* wild-type cells upon exposure to: **(A)** *G. mellonella* hemocytes, comparing non-internalized cells collected in the supernatant of hemocyte-*C. glabrata* 1 h co-cultures, and after 24 and 48 h of internationalization by hemocytes; **(B)** 30, 60, or 100 mM of acetic acid for 1 h; or **(C)** 20 mM of hydrogen peroxide for 1 h, in comparison with exponentially growing *C. glabrata* cells. The presented transcript levels were obtained by quantitative RT-PCR and are *CgDTR1mRNA/CgACT1*mRNA levels, relative to the values registered in wild-type exponential cells cultivated in planktonic conditions. The indicated values are averages of at least three independent experiments. Error bars represent the corresponding standard deviations. ^*^P < 0.05; ^***^P < 0.001; ^****^P < 0.0001.

Considering the importance of CgDtr1 for proliferation within hemocytes, its transcript levels were followed during internalization in hemocytes. A dramatic up-regulation of *CgDTR1* expression was registered during the internalization of *C. glabrata* cells by *G. mellonella* hemocytes (Figure 7A). Interestingly, upon 1 h of co-culture with hemocytes, *C. glabrata* cells that were not internalized by hemocytes already exhibit a 4-fold up-regulation of *CgDTR1* transcript levels. Upon 24 or 48 h of internalization of *C. glabrata* cells in hemocytes the transcript levels of *CgDTR1* were found to be 100-fold up-regulated, which highlights the importance of this gene in the context of the adaptation of this pathogen to growth inside macrophages. In order to evaluate which could be the stimuli that leads to this up-regulation, changes in *CgDTR1* transcript levels occurring in *C. glabrata* cells upon 1 h of exposure to either acetic acid or hydrogen peroxide were assessed. Of the two stresses only hydrogen peroxide was found to induced a significant up-regulation of *CgDTR1* expression, while surprisingly exposure to acetic acid led to the down-regulation of *CgDTR1* expression (Figures 7B,C).

## Discussion

In this study, the multidrug transporter CgDtr1 was shown to play a role in *C. glabrata* pathogenesis, protecting these cells from stress agents present in macrophagic cells.

The deletion of *CgDTR1* was found to lead to an increased larvae survival upon infection, decreasing in 30% the killing ability of *C. glabrata* cells. The use of *G. mellonella* as a model, had been previously exploited for the study of *Candida albicans* (Jacobsen, 2014) and *C. glabrata* (Santos et al., 2017) virulence, and appears to be particularly useful in this context since it is difficult to cause *C. glabrata*-induced mortality in mice, even when facing neutropenia (Jacobsen et al., 2010). Furthermore, the larval innate immune system has a strong similarity to the mammalian innate immune system, especially when considering the hemocyte-dependent cellular response, very similar to that displayed by mammalian macrophages (Ribeiro and Brehélin, 2006), in which *C. glabrata* is able to survive and replicate for a very prolonged time (Seider et al., 2011). These features make the use of *G. mellonella* larvae an interesting and simplified model to further understand what happens inside the human host.

Although previous reports had shown that some *Candida* DHA genes (Yamada-Okabe and Yamada-Okabe, 2002; Shah et al., 2014; Santos et al., 2017), homologs of *CgDTR1*, play a role in virulence, the exact mechanisms underlying these observations remained unclear. In this work, the deletion of the *CgDTR1* gene was found to decrease the ability of *C. glabrata* to kill *G. mellonella* larvae. This decreased killing ability was found to correlate with diminished proliferation in *G. mellonella* hemolymph. Our results of *in vitro* interaction between yeast cells and hemocytes seem to support the hypothesis that CgDtr1 contributes for cell proliferation within macrophages. It is clear that Δ*cgdtr1* cells are more susceptible to stress factors found inside phagocytic cells, including acidic and oxidative stress. Additionally, the dramatic up-regulation of *CgDTR1* transcript levels in *C. glabrata* cells upon internalization in *G. mellonella* hemocytes, which correlates with increased *CgDTR1* expression induced upon exposure to oxidative stress, further suggests that CgDtr1 is an important factor for adaptation to proliferate inside host macrophages.

Consistent with a role in weak acid stress resistance, CgDtr1 was found in this study to be a plasma membrane acetate exporter. Weak acids have an impact on membrane organization and function, while their intracellular accumulation may lead to higher oxidative stress, protein aggregation, membrane trafficking inhibition and changes in plasma and vacuolar membrane spatial organization (Piper et al., 2001; Teixeira et al., 2007). The expression of a number of genes encoding DHAs have been found to contribute to weak acid tolerance in *S. cerevisiae*, including *AQR1* and *AZR1* (Tenreiro et al., 2000, 2002), *TPO2* and *TPO3* (Fernandes et al., 2005) and *TPO1* (Godinho et al., 2017). Interestingly, CgDtr1 shares with its *S. cerevisiae* homolog Dtr1 a role in weak acid stress tolerance. However, given its plasma membrane localization and the inability of *C. glabrata* to undergo sporulation, it is unlikely that CgDtr1 shares Dtr1's role as dityrosine transporter in *S. cerevisiae* prospore membrane (Felder et al., 2002). Tolerance against weak acids is important for at least *C. albicans*, as well as probably other pathogenic *Candida* species, to thrive within the host, since significant concentrations of both acetic and lactic acids have been found in the vaginal tract (Davis, 2009), and within the phagolysosome of human macrophages (Peleg et al., 2010). Furthermore, a synergistic action between some drugs and acetic acid was found, reinforcing the importance of weak acid resistance mechanisms in the context of antifungal therapy (Costa et al., 2013a).

Altogether, the results from the present study strongly suggest that CgDtr1 plays an important role in the virulence of *C. glabrata* infections, possibly as a fitness factor (Kasper et al., 2015), facilitating its proliferation within the host by protecting against weak acid stress.

## Author contributions

DR and MC made most of the experimental work and drafted the manuscript. RS, PP and CC contributed to the experimental work. DM-H and AF designed and conducted the infection experiments. AT-N and HC designed and conducted the molecular biology of *C. glabrata* strains. HC is a co-corresponding author of this manuscript. MT designed the experimental workflow, supervised the experimental work, and wrote the manuscript.

### Conflict of interest statement

The authors declare that the research was conducted in the absence of any commercial or financial relationships that could be construed as a potential conflict of interest.
